# Factors relating to eating style, social desirability, body image and eating meals at home increase the precision of calibration equations correcting self-report measures of diet using recovery biomarkers: findings from the Women’s Health Initiative

**DOI:** 10.1186/1475-2891-12-63

**Published:** 2013-05-16

**Authors:** Yasmin Mossavar-Rahmani, Lesley F Tinker, Ying Huang, Marian L Neuhouser, Susan E McCann, Rebecca A Seguin, Mara Z Vitolins, J David Curb, Ross L Prentice

**Affiliations:** 1 Department of Epidemiology & Population Health, Division of Health Promotion & Nutrition Research, Albert Einstein College of Medicine, Bronx, NY, 10461, USA; 2 Division of Public Health Sciences, Fred Hutchinson Cancer Research Center, Seattle, WA, 98109-1024, USA; 3 Division of Cancer Prevention and Population Sciences, Roswell Park Cancer Institute, Buffalo, Elm & Carlton Streets, NY, 14263, USA; 4 Division of Nutritional Sciences, Cornell University, Ithaca, NY, 14853, USA; 5 Department of Epidemiology & Prevention, Wake Forest University Health Sciences, Winston-Salem, NC, 27157-1063, USA; 6 Department of Geriatric Medicine, University of Hawaii, Honolulu, HI, USA

**Keywords:** Measurement error, Dietary assessment, Psychosocial instruments, Dietary behavior, Four day food record, Food frequency questionnaire, 24 hour dietary recall

## Abstract

**Background:**

The extent to which psychosocial and diet behavior factors affect dietary self-report remains unclear. We examine the contribution of these factors to measurement error of self-report.

**Methods:**

In 450 postmenopausal women in the Women’s Health Initiative Observational Study doubly labeled water and urinary nitrogen were used as biomarkers of objective measures of total energy expenditure and protein. Self-report was captured from food frequency questionnaire (FFQ), four day food record (4DFR) and 24 hr. dietary recall (24HR). Using regression calibration we estimated bias of self-reported dietary instruments including psychosocial factors from the Stunkard-Sorenson Body Silhouettes for body image perception, the Crowne-Marlowe Social Desirability Scale, and the Three Factor Eating Questionnaire (R-18) for cognitive restraint for eating, uncontrolled eating, and emotional eating. We included a diet behavior factor on number of meals eaten at home using the 4DFR.

**Results:**

Three categories were defined for each of the six psychosocial and diet behavior variables (low, medium, high). Participants with high social desirability scores were more likely to under-report on the FFQ for energy (β = -0.174, SE = 0.054, *p* < 0.05) and protein intake (β = -0.142, SE = 0.062, *p* < 0.05) compared to participants with low social desirability scores. Participants consuming a high percentage of meals at home were less likely to under-report on the FFQ for energy (β = 0.181, SE = 0.053, *p* < 0.05) and protein (β = 0.127, SE = 0.06, *p* < 0.05) compared to participants consuming a low percentage of meals at home. In the calibration equations combining FFQ, 4DFR, 24HR with age, body mass index, race, and the psychosocial and diet behavior variables, the six psychosocial and diet variables explained 1.98%, 2.24%, and 2.15% of biomarker variation for energy, protein, and protein density respectively. The variations explained are significantly different between the calibration equations with or without the six psychosocial and diet variables for protein density (p = 0.02), but not for energy (p = 0.119) or protein intake (p = 0.077).

**Conclusions:**

The addition of psychosocial and diet behavior factors to calibration equations significantly increases the amount of total variance explained for protein density and their inclusion would be expected to strengthen the precision of calibration equations correcting self-report for measurement error.

**Trial registration:**

ClinicalTrials.gov identifier: NCT00000611

## Background

Food frequency questionnaires (FFQs) have been used extensively in nutritional epidemiology research. Other approaches include the 24 hour dietary recall (24HR) and the four day food record (4DFR). These self-report measures include systematic and random errors that can distort associations between diet and disease [1]. Calibration equations that adjust for systematic and random aspects of self-report measurement error provide a methodology for correcting diet and disease association estimates. Using this approach Prentice et al. report that biomarker calibrated, but not uncalibrated energy is positively correlated with total and site-specific cancer incidence [1] and coronary heart disease incidence [2] while Tinker et al. note corresponding findings for calibrated, but not uncalibrated protein intake in relation to diabetes risk [3].

The addition of readily available participant characteristics such as body mass index, age and ethnicity to the calibration equations in Women’s Health Initiative (WHI) biomarker studies has further enhanced the ability to explain much larger fractions of biomarker variation than self-report estimates alone [4,5]. However there is a paucity of research on participant behaviors that might impact self-report such as social desirability [6], body image, emotional, uncontrolled or restrained eating and eating more meals at home. Social desirability is the tendency of respondents to answer questions in a manner that will be viewed favorably by others; restrained eating is the conscious effort to restrict calorie intake, and uncontrolled eating is the loss of self-control in eating behavior when faced with anxiety and distress [7,8]. Social desirability may impact self-report by over-reporting of “favorable” and under-reporting of “unfavorable” foods. In collectivist societies, there may be a greater need to respond in a socially sanctioned way to maintain good relationships and save face as compared with individualistic societies where honesty in interactions with strangers is a characteristic that is more highly valued [9].

The wealth of biomarker and psychosocial data collected in the WHI biomarker studies provides a unique and special opportunity to assess the contribution of psychosocial variables to self-report measures of diet as reflected in their ability to explain biomarker variation for energy, protein and protein density. Given the heterogeneity within study populations and presence of newly established immigrants in our study sample, exploration of additional participant characteristics such as psychosocial and diet behavior factors that may impact self-report and possibly strengthen calibration equations is strongly merited.

Data from previous studies indicate that under-reporting in women is associated with fear of negative evaluation, weight loss history, percentage of energy from fat and eating less frequently or variability in number of meals per day [10,11]. Under-reporting of energy intake has been found in both older and younger participants [5,10-12] and under-reporters tend to be less physically active, more likely to diet and eat less fat as a percentage of energy intake compared with accurate reporters [13]. Other investigators found that reporting accuracy in food records was significantly associated with social desirability and body size dissatisfaction in women [14-16]. A study using the WHI FFQ indicated that women who perceive themselves to be thin according to the Stunkard-Sorenson silhouettes were more likely to under-report energy intake than women who perceived themselves to be heavy [16]. Additional factors associated with under-reporting included restrained eating or the conscious effort to restrict calorie intake [7,8] and high disinhibition or the loss of self-control in eating behavior when faced with anxiety and distress [7,8]. Here we examine the extent to which psychosocial and diet behavior factors affect self-report in the Women’s Health Initiative-Nutrition & Physical Activity Assessment Study (WHI-NPAAS) and how they can augment calibration equations, for each of the FFQ, 4DFR and 24HR assessment methodologies.

## Methods

This research was conducted to investigate the ability to augment biomarker-calibrated self-reports for dietary intakes of energy, protein and protein density by adding measures of social desirability, body image eating factors and a measure of dietary behavior.

### Study population

Details for the (WHI-NPAAS) in which participants for this study were enrolled have been published previously [4]. Briefly, the WHI Observational Study is a prospective cohort study that enrolled 93,676 postmenopausal women in the age range 50–79 years during 1994–1998 at 40 US clinical centers [17,18]. Four hundred and fifty postmenopausal women from the WHI Observational Study were enrolled in the WHI-NPAAS from 2007–2009. This sample size of 450 was chosen based on extensive computer simulations to provide effective calibration and to yield hazard ratio estimators of acceptable precision when calibrated consumption estimates are used in disease association studies. To support comparisons of measurement properties among sub-groups in the WHI-NPAAS, three groups of women were oversampled. These were Black and Hispanic women, younger post-menopausal women and women at high and low ends of BMI distribution. Women were excluded for having any medical condition precluding participation, weight instability, or travel plans during the study period. Overall, 20.6% of women who were invited and screened for eligibility completed the protocol. This 20.6% recruitment rate, in part reflects reaching the enrollment goal before exhausting the recruitment list which was built to support the enrollment process. Data on similarities and differences between this sub-sample and the WHI Observational Study cohort have been published previously [4]. An additional 4 women consented to, but did not complete the study. A sub-sample of 88 women (19.6%) repeated the entire protocol approximately 6 months later to provide repeatability information. Study procedures were approved by the institutional review boards of participating institutions and informed consent was obtained from participants.

### Study protocol & procedures

#### Biomarker assessment

**Figure 1:**
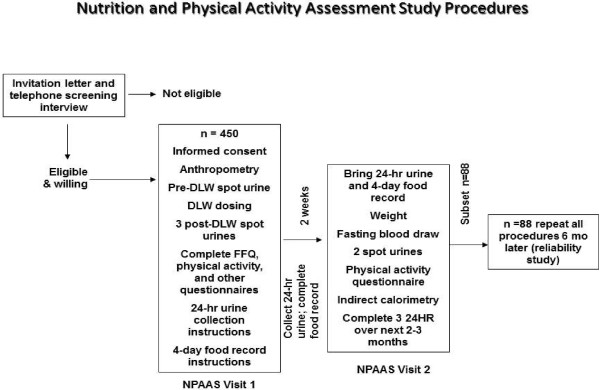
**Ross L. Prentice, Mossavar-Rahmani et al. Evaluation and comparison of food records, recalls, and frequencies for energy and protein assessment by using recovery biomarkers *Am. J. Epidemiol. (2011) 174(5): 591-603, Fig. 1.***

Study protocol consisted of two visits with in-home activities between the visits (see Figure 1). Doubly labeled water (DLW) was administered at visit one. Four timed spot urines were collected at visit one: one at baseline and three post baseline. At home between the visits, participants collected 24HR urine the day before the second visit, which occurred two weeks later. At the second clinic visit, women provided two more timed urine collections, fasting blood draw and completed indirect calorimetry. DLW and urinary nitrogen were used as biomarkers of objective intake for total energy and protein consumption respectively. They were compared against self-report using three dietary assessment tools: FFQ, 4DFR, and 24HR.

#### Dietary assessment

All dietary assessments (FFQ, 4DFR, 24HR) were conducted in English or Spanish as appropriate. All questionnaires (including psychosocial questionnaires) were translated into Spanish and Spanish translations were back-translated into English. The WHI-FFQ was collected at the first of two study visits and participants recorded 4DFR at home between the study visits. Three 24HR were collected at monthly intervals three months after the two visits were completed. The 24HR were conducted by trained and certified study staff by telephone with data entered directly and computerized by using the Nutrition Data System for Research (NDSR); Nutrition Coordinating Center, University of Minnesota, Minneapolis, Minnesota software.

### Psychosocial factors

At the first study visit psychosocial data were collected. These included the Stunkard-Sorenson Body Silhouettes for body image perception; the Crowne-Marlowe Social Desirability Scale for social desirability, and the Three Factor Eating Questionnaire (R-18) for assessing cognitive restraint for eating, uncontrolled eating, and emotional eating.

#### Stunkard-Sorenson body silhouettes

These silhouettes consisted of drawings of 9 different female body shapes of increasing body size from very thin to very fat [19,20]. These images have been widely used in epidemiological investigation and represent an easy-to-administer self-report measure of body image. The participants were asked which figure reflects: “how you think you look; how you feel most of the time; is your ideal figure (for you); you think is ideal for women; you think is preferred by men.” Differences between each participant’s perceived current body silhouette and what she perceived as healthy, ideal were computed.

#### Crowne-Marlowe Social Desirability Scale

The Crowne-Marlowe Social Desirability Scale consists of 33 true-false items: a higher score indicates greater social desirability [6]. Social desirability is the tendency to respond to questionnaires or interviews with what is perceived to be a socially appropriate response as opposed to an objective or accurate response. This scale has been shown to be internally consistent (Kuder-Richardson formula 20 coefficient = 0.88) and to have good test-retest reliability (r = 0.89) [6].

#### Three Factor Eating Questionnaire

We used a revised 18-item version of the Three Factor Eating Questionnaire (TFEQ-R18) that measures three aspects of eating behavior: cognitive restraint of eating, uncontrolled eating and emotional eating [21]. The reliability of factors has been measured using Cronbach’s alpha values which in pooled data were 0.79 for cognitive restraint, 0.82 for uncontrolled eating and 0.89 for emotional eating [22]. The 18 items are on a 4 point response scale: definitely true/mostly true/mostly false, and definitely false. Responses to each of the 18 items are given a score between 1 and 4 and item scores are summated into scale scores for cognitive restraint, uncontrolled eating and emotional eating. Higher scores in the respective scales indicate greater cognitive restraint, uncontrolled or emotional eating.

#### Meals at home

Because the FFQ does not include eating location information, data on eating location of meals were only derived from the 4DFR and the 24HR. Percent of meals eaten at home was calculated for each participant. Since more days were recorded with the 4DFR compared to the 24 HRs, we based our analyses on “meals at home” on 4DFR data.

### Statistical methods

Our objective was to determine whether psychosocial factors and dietary behavior were associated with the biases in self-reported dietary assessment tools and whether the addition of psychosocial factors and dietary behavior improved the calibration equations that account for measurement error of self-reported dietary assessment tools. These analyses focused on log-transformed consumption estimates for each of energy, protein and protein density, which were each approximately normally distributed [5]. In weight stable persons, urinary recovery of metabolites produced when energy and protein are expended leads to objective estimates of short-term energy and protein consumption [3]. Outliers with values outside of the interquartile range by more than three times its width were excluded from analysis. Calibration equations for use in disease risk association studies were developed using linear regression models that predicted true intakes of energy and protein given the self-reported intakes and data on study subject characteristics based on the following measurement error models.

First, we assume a log (biomarker) assessment *W* adheres to a classical measurement model,

(A) W=Z+e

where *Z* is the targeted nutritional variable, and *e* is an independent error term that is assumed to be independent of *Z* and other study subject characteristics. *Z* can be regarded as the logarithm of average daily consumption for the nutritional factor under study over a fairly short period of time such as 6–12 months in proximity to the biomarker data collection period. Second, for self-reported dietary assessment tools and psychosocial and diet behavior factors, the following expanded and more flexible measurement model was considered:

(B) $$Q=S_{o}+S_{1}Z+S_{2}V+S_{3}\mathrm{VZ}+r+u$$

for the (log-transformed) self-report nutrient assessment *Q*, where, *S*_*0*_, *S*_*1*_, *S*_*2*_, and *S*_*3*_ are regression parameters to be estimated, V is a set of characteristics that may relate to systematic bias in the assessment (such as body mass index (BMI), race/ethnicity, and age in addition to the psychosocial characteristics), *r* is a person-specific error variable, and *u* is an independent measurement error term. Also r and u are independent of Z, V, and e.

In our analyses, we first examined the correlation between the psychosocial and dietary variables, including the body image variable, the TFEQ-R18, and the diet behavior about percent meals eaten at home from 4DFR/24HR. Next, these psychosocial and dietary variables were entered into a linear regression model in addition to age, body mass index, and ethnicity, for association with the difference between log (self-reported nutrient) and log (biomarker) (Q-W). Finally, we conducted a series of linear regression of log (biomarker) Won log(self-report nutrient) Q and participant characteristics V including age, body mass index, ethnicity, psychosocial and dietary behavior factors. Based on our measurement error models these calibration equations allow estimation of targeted nutritional value Z based on Q and V. We calculated the fraction of the total variance in the log-transformed biomarker (R^2^) that could be explained by the self-report assessment and participant characteristics. Let W_1_ = Z + e_1_ and W_2_ = Z + e_2_ be the primary and reliability biomarker measures of the same individual, we also calculated the adjusted R^2^ values as R^2^ value divided by corr(W_1_, W_2_) –0.5ρ × var(W_1_-W_2_)/(1- ρ)/var(W), where ρ = corr(e_1_,e_2_) is the correlation between the measurement errors for W_1_ and W_2_[23]. The denominator corresponds to the ratio of the variance of Z relative to the variance of W given our measurement error model for W. Consequently the adjusted R^2^ can be interpreted as the percentage of variation in Z explained by Q and V in the calibration model. Since the correlation ρ cannot be estimated based on the available data, we conducted sensitivity analysis exploring the explained variation in underlying Z for varying ρ. In all the regression models, we categorized the psychosocial factors into the low, medium, and high categories, based on <1, 1–2, and > = 3 for body image and tertiles for other psychosocial factors.

With regard to social desirability this cohort scored highly positively and only 4 participants scored < 9, consequently we had too few in the group considered low to differentiate this group from medium and high based on the Crowne-Marlowe classification of <9 as low; 9–19 as medium and 20–33 [6]. As a result our low, medium and high scores for social desirability are based on tertiles and the cut-off for high scores are much higher than those of Crowne and Marlowe [6]. Specifically, the low, medium, high categories represent scores < =79.6, 79.6-92.3, >92.3 for percent of meals eaten at home, <=19, 20–24, >24 for social desirability, <=14, 15–16, >16 for cognitive restraint, <=24, 25–27, >27 for uncontrolled eating, and < =7, 8–10, >10 for emotional eating.

For energy, protein and protein density, we examined the incremental value of adding psychosocial and dietary behavior factors into the regression model by comparing the R^2^ between the simpler models with age, body mass index, and ethnicity and the model with psychosocial factors added. Bootstrap procedure was used for estimating standard errors for these comparisons based on 5,000 bootstrap samples. All statistical procedures were conducted using statistical software R version 2.14.1 (http://cran.r-project.org).

## Results

Table 1 shows the distribution of demographic and background characteristics for 450 NPAAS participants. The sample is highly educated; with more than half having a college education. Thirty-eight percent are obese.

**Table 1:** Characteristics of NPAAS participants in primary sample based on NPAAS primary visit

**Characteristic**	**N = 450**	**%**
	**(Median, inter-quartile range if applicable)**	
**Age (years)**		
59-69	213	47.3
69-79	190	42.2
79-91	47	10.4
**Body Mass Index**		
<25	156	34.7
25- < 30	121	26.9
> = 30	173	38.4
**Race/Ethnicity**		
Non-Hispanic White	228	64.0
African-American	84	18.6
Hispanic	64	14.2
Asian/Pacific Islander	8	1.8
Other	6	1.3
**Annual income (dollars $)**		
<20,000	43	9.9
20,000-34,999	92	21.2
35,000-49,999	84	19.4
50,000-74,999	98	22.6
> = 75,000	117	27.0
**Education**		
College degree or higher	226	50.6
Some college	157	35.1
High school diploma/GED	48	10.7
Less than high school	16	3.6
**Current Smoking**	11	2.5
**Recreational episodes per week:**		
<2	70	15.8
2-4	36	8.1
>4	337	76.1
**% Meals Eaten at Home:**		
Based on the Four Day Food Record (4DFR)	85.7 (76.5-94.1)	
Based on the 24 Hr Dietary Recall (24HR)	81.8 (68.8-92.3)	
**Body Image:**		
Discordance (perceived minus ideal)	1.0 (1.0-2.0)	
**Three Factor Eating Scale (TFEQ-R18) **^**1**^**:**		
Cognitive Restraint of Eating	15.0 (14.0-16.0)	
Uncontrolled Eating	26.0 (23.0-28.0)	
Emotional Eating	9.9 (6.0-11.0)	
**Crowne-Marlowe Social Desirability **^**2**^	21.0 (17.0-25.0)	

^1^possible values from 6–24 for Restraint, 9–36 for Uncontrolled Eating, and 3–12 for Emotional Eating.

^2^possible values from 0 to 33.

### Body image

Participants selected an average perceived body size (feel) of 4.87 (SD = 1.49); versus 4.92 (SD = 1.40) for perceived body size (think). Of the nine sizes, the mean size selected or 4.92 is slightly higher than the middle of the range from very thin to very fat. Mean difference between perceived body size think versus feel was insignificant at mean 0.06 (SD = 0.94) with P = 0.192. Body image discordance that is the difference between perceived and ideal for *self* was somewhat modest at 1.25 (SD = 1.06) with P < 0.001; a slightly higher level of discord was evident in the difference between “perceived and ideal for *women*”: 1.45 (SD = 1.36) with P < 0.001 and an even higher level in difference between “perceived and ideal for *men*”: 1.87 (1.61) with P < 0.001. For the purposes of this research, we focused our analyses on body image discordance defined as difference between “perceived and ideal for *self*” as opposed to ideal for women or men.

### Three Factor Eating Questionnaire

The mean (SD) scores for the TFEQ-R18 were: 15.24(1.98) for cognitive restraint, 25.66(3.47) for uncontrolled eating and 8.47(2.6) for emotional eating. Higher body mass index was associated with lower level of uncontrolled eating (Pearson correlation r = -0.27 with P < 0.001) and emotional eating (Pearson correlation -0.40 with P < 0.001), but not with cognitive restraint (r = 0.06 with P = 0.226).

### Dietary behavior: meals at home

Based on 4DFR and 24HR recall respectively, on average 83.0% and 78.6% of meals were consumed at home.

### Social desirability

Sixty percent of NPAAS women scored high, 39% as medium and 0.9% as low according to the Crowne-Marlowe Scale classification (20–33 as high, 9–19 as medium and <9 as low), with a mean (SD) social desirability score of 21.07 (5.35). We present the median and inter-quartile range in Table 1 and our classification of low, medium, high in the tables that follow are based on tertiles.

### Inter-correlations between the psychosocial factors and dietary behavior

Significant, but modest positive correlations were observed between cognitive restraint and uncontrolled eating (r = 0.15, P = 0.001), cognitive restraint and emotional eating (r = 0.14, P = 0.002); the correlation of uncontrolled eating and emotional eating was stronger (r = 0.60, P < 0.001) suggesting substantive association between these domains.

Women with high social desirability scores had significantly higher scores for uncontrolled eating (r = 0.25, P < 0.001) and emotional eating (r = 0.29, P < 0.001). Women with high body image discordance had lower scores for cognitive restraint of eating (r = -0.35, P < 0.001), but also lower level of emotional eating (r = -0.38, P < 0.001). Women who ate more meals at home had higher scores for uncontrolled eating than women who ate fewer meals at home (r = 0.096, P = 0.043).

### Associations with reporting error for energy, protein and % energy from protein

Tables 2, 3, 4 show the estimates of the regression coefficients β and their standard errors from the regression of log(self-report) minus log(biomarker) on BMI, age, ethnicity, psychosocial and dietary variables for energy, protein and % energy from protein (protein density). Each of the dietary self-report instruments shows evidence of systematic bias related to one or more of the factors mentioned above.

**Table 2:** Regression of log(self-report) minus log(biomarker) on predictors in NPAAS (n = 450) for energy

	**Food frequency questionnaire**		**4-day food record**		**24-hour dietary recall**	
**Variable**	**β**	**SE**	**β**	**SE**	**β**	**SE**
Intercept	-0.5811	0.303	-0.2765	0.183	-0.1826	0.208
BMI	-0.0038	0.004	-0.0113*	0.003	-0.016*	0.003
Age	0.0072*	0.004	0.0064*	0.002	0.0062*	0.003
Black	-0.2532*	0.06	-0.0473	0.036	-0.0487	0.041
Hispanic	-0.1789*	0.064	-0.0042	0.039	-0.0277	0.044
Other	-0.2183	0.117	-0.0536	0.07	-0.1028	0.08
Meals at home-M	0.0855	0.051	-0.0304	0.031	-0.0102	0.035
Meals at home-H	0.1814*	0.053	-0.0121	0.032	0.0185	0.036
Body Image-M	0.009	0.062	0.0277	0.038	0.0286	0.043
Body Image-H	-0.0808	0.102	-7.00E-04	0.061	0.0442	0.07
TFEQ-R18						
Restraint-M	-0.0806	0.048	-0.0264	0.029	-0.0299	0.033
Restraint-H	-0.0159	0.058	-0.0371	0.035	0.0155	0.04
Unc. Eat-M	-0.0991	0.054	-0.0234	0.033	-0.0043	0.037
Unc. Eat-H	-0.0831	0.063	-0.0248	0.038	-0.0127	0.043
Emo. Eat-M	-0.0154	0.054	-0.0338	0.033	-0.0423	0.037
Emo. Eat-H	0.0658	0.074	0.0332	0.044	-0.0116	0.051
Soc. Des-M	-0.0584	0.05	-0.0431	0.03	-0.0548	0.034
Soc. Des-H-	-0.1744*	0.054	-0.043	0.032	-0.0549	0.037

Psychosocial & meals at home variables: medium and high are compared to low.

Meals at home based on 4DFR.

*Beta coefficient differs from zero at the 0.05 level of significance.

**Table 3:** Regression of log(self-report) minus log(biomarker) on predictors in NPAAS (n = 450) for protein

	**Food frequency questionnaire**		**4-day food record**		**24-hour dietary recall**	
**Variable**	**β**	**SE**	**β**	**SE**	**β**	**SE**
Intercept	-0.6274	0.349	-0.3345	0.217	-0.5412*	0.229
BMI	0.002	0.005	-0.0064*	0.003	-0.0099*	0.003
Age	0.0087*	0.004	0.0074*	0.003	0.0104*	0.003
Black	-0.199*	0.07	0.0944*	0.044	0.1163*	0.046
Hispanic	-0.1099	0.073	0.0341	0.045	0.0417	0.048
Other	-0.1049	0.135	-0.0588	0.084	0.0134	0.088
Meals at home-M	0.0161	0.059	-0.0621	0.037	-0.0365	0.039
Meals at home-H	0.1268*	0.06	-0.0182	0.037	-0.0024	0.039
Body Image-M	-0.0111	0.071	-0.0071	0.044	0.0295	0.046
Body Image-H	-0.1688	0.115	-0.1097	0.072	-0.0283	0.076
TFEQ-R18						
Restraint-M	-0.0263	0.055	0.0157	0.034	-0.004	0.036
Restraint-H	-0.0083	0.068	-0.0169	0.042	-0.0471	0.045
Unc. Eat-M	-0.0735	0.062	0.0263	0.039	0.0023	0.041
Unc. Eat-H	-0.047	0.072	0.0052	0.045	0.0186	0.048
Emo. Eat-M	-0.0342	0.062	-0.0678	0.039	-0.0475	0.041
Emo. Eat-H	0.0265	0.084	0.0416	0.052	0.0083	0.055
Soc. Des-M	-0.0335	0.058	0.0027	0.036	-0.0361	0.038
Soc. Des-H-	-0.1423*	0.062	-0.0528	0.039	-0.0434	0.041

Psychosocial & meals at home variables: medium and high are compared to low.

Meals at home based on 4DFR.

*Beta coefficient differs from zero at the 0.05 level of significance.

**Table 4:** Regression of log(self-report) minus log(biomarker) on predictors in NPAAS (n = 450) for protein density

	**Food frequency questionnaire**		**4-day food record**		**24-hour dietary recall**	
**Variable**	**β**	**SE**	**β**	**SE**	**β**	**SE**
Intercept	-0.0298	0.237	0.0944	0.218	-0.3049	0.228
BMI	0.0063	0.003	0.0047	0.003	0.0037	0.003
Age	0.0013	0.003	-3.00E-04	0.003	0.0045	0.003
Black	0.0679	0.047	0.1307*	0.043	0.152*	0.045
Hispanic	0.1103*	0.05	0.0544	0.046	0.0932	0.048
Other	0.0832	0.09	-0.0123	0.083	0.1105	0.086
Meals at home-M	-0.0474	0.04	-0.0347	0.036	-0.0198	0.038
Meals at home-H	-0.0316	0.041	-0.013	0.037	-0.0064	0.039
Body Image-M	-0.0339	0.048	-0.0656	0.044	-0.0079	0.046
Body Image-H	-0.118	0.079	-0.1035	0.073	-0.0346	0.076
TFEQ-R18						
Restraint-M	0.038	0.037	0.0318	0.034	0.0271	0.036
Restraint-H	0.0076	0.046	0.0023	0.042	-0.0472	0.044
Unc. Eat-M	0.0086	0.042	0.0511	0.039	0.0296	0.041
Unc. Eat-H	0.0366	0.049	0.0311	0.045	0.0745	0.047
Emo. Eat-M	-0.0412	0.043	-0.0473	0.039	-0.0481	0.041
Emo. Eat-H	-0.057	0.057	-0.0013	0.053	-0.0232	0.055
Soc. Des-M	0.0365	0.039	0.053	0.036	0.0334	0.037
Soc. Des-H-	0.0146	0.042	-0.0154	0.038	-0.0254	0.04

Psychosocial & meals at home variables: medium and high are compared to low.

Meals at home based on 4DFR.

*Beta coefficient differs from zero at the 0.05 level of significance.

Here we report significant results relating to the psychosocial and diet variable (meals at home) with significance level set at 0.05. The FFQ systematic bias patterns included substantially greater underestimation among women with high social desirability scores compared to those with low social desirability scores, both for total energy and for protein intake. On the other hand, women who consumed high percentage of meals at home were less likely to underestimate energy or protein intake compared to women with low percentage of meals consumed at home.

### Psychosocial variables and attendant increase in R^2^

In Tables 5, 6, 7, 8, we show regression coefficients estimates and standard errors in the calibration equation and the associated R^2^ for energy, protein, and protein density separately. Results are presented for calibration equations including each individual dietary assessment separately and calibration equations including FFQ, 4DFR and 24HR together. In general psychosocial and dietary variables appear to account for a modest variability in biomarker measure (less than 5%) after age, BMI, and race were already adjusted for. Table 8 shows R^2^ results for the calibration equations that include FFQ, 4DFR, and 24HR together as well as age, BMI, race, and all psychosocial variables. In the calibration equations including self-report assessments together with age, BMI, race, and the psychosocial and dietary behavior factors, the six psychosocial and dietary factors explained 1.98%, 2.24% and 2.15% of biomarker variation for energy, protein, and protein density respectively. The variation explained by calibration equations in Table 8 are significantly different from the variation explained by the calibration equations without the six psychosocial and diet variables for protein density (p = 0.03), but not for energy (p = 0.119) or protein intake (p = 0.077). In the calibration equations including 4DFR, age, BMI, race and the psychosocial and dietary variables, the six psychosocial and dietary variables explained 2.17%, 2.72%, 2.66% biomarker variation for energy, protein, and protein density respectively (Tables 5, 6, 7). Statistically significant differences were observed between the 4DFR calibration equations with or without the six psychosocial and dietary variables for both protein intake (p = 0.027) and protein density (p = 0.017), but not for energy (p = 0.075). Finally, for Z defined in terms of a short period of time such as six months, we consider negative correlations between biomarker measurement errors of the primary and reliability data for calculating the adjusted R^2^, since the diets during the primary and reliability data collection periods may differ more than is the case for other short periods during this 6 month interval. The corresponding adjusted R^2^ for calibration equations shown in Table 8 were 75.3%, 64.8%, and 79.1% for energy, protein, and protein density respectively assuming a negative correlation of -0.1, with adjusted variation explained by psychosocial and dietary behavior factors being 3.11%, 3.92%, and 8.63% respectively. Assuming a negative correlation of -0.2, the corresponding adjusted R^2^ for calibration equations shown in Table 8 were 72.2%, 59.6%, and 62.5% for energy, protein, and protein density respectively, with adjusted variation explained by psychosocial and dietary behavior factors being 2.99%, 3.61% and 6.82% respectively.

Tables 5, 6, 7 and 8 in this section show regression coefficients from linear regression of log (biomarker) on log (self-report), as well as body mass index, age, ethnicity and the psychosocial and dietary variables for energy, protein and protein density, thereby allowing an adjustment for the systematic biases from Tables 2, 3 and 4.

**Table 5:** **Energy, regression of log(biomarker) on log(self-report) and other predictors in NPAAS (n = 450) and R**^**2**^

	**Food frequency questionnaire (FFQ)**			**4 day food record**			**24 hour dietary recall**		
**Variable**	**β**	**SE**	**R**^**2**^	**β**	**SE**	**R**^**2**^	**β**	**SE**	**R**^**2**^
Intercept	7.6551*	0.029		7.6260*	0.028		7.6397*	0.028	
Log(FFQ- Calories-C)	0.0459*	0.018	3.624	0.1559*	0.029	8.381	0.0921*	0.027	3.056
BMI-C	0.0136*	0.002	27.847	0.0136*	0.001	27.729	0.0137*	0.001	29.497
Age-C	-0.0092*	0.001	9.485	-0.0089*	0.001	8.257	-0.0089*	0.001	8.786
Race:			1.405			1.420			1.441
Black	-0.0298	0.021		-0.0292	0.020		-0.0303	0.020	
Hispanic	-0.0609*	0.023		-0.0604*	0.022		-0.0595*	0.022	
Other	-0.0448	0.040		-0.0401	0.039		-0.0413	0.040	
Meals at home:			0.628			0.282			0.364
Med	-0.0332	0.017		-0.0212	0.017		-0.0239	0.017	
High	-0.0151	0.018		-0.0041	0.018		-0.0054	0.018	
Body Image:			0.375			0.369			0.356
Med	-0.0275	0.021		-0.0283	0.021		-0.0267	0.021	
High	-0.0528	0.035		-0.0499	0.034		-0.0504	0.034	
TFEQ-R18:									
Restraint:			0.337			0.302			0.237
Med	0.0251	0.017		0.0231	0.016		0.0207	0.016	
High	0.0295	0.020		0.0312	0.019		0.0267	0.020	
Unc. Eat.:			0.795			0.666			0.819
Med	-0.0286	0.019		-0.0256	0.018		-0.0289	0.018	
High	-0.0296	0.022		-0.0257	0.021		-0.0298	0.021	
Emo. Eat.:			0.328			0.428			0.372
Med	0.0087	0.019		0.0123	0.018		0.0121	0.018	
High	-0.0217	0.025		-0.0217	0.025		-0.0185	0.025	
Social Desirability:			0.075			0.124			0.155
Med	0.0117	0.017		0.0147	0.017		0.0160	0.017	
High	0.0026	0.019		0.0019	0.018		0.0021	0.018	
Total^1^			44.897			47.959			45.084

BMI, body mass index.

^*^β Coefficient differs from zero at p = 0.05 significance level.

^1^Total – percent of variation explained by all variables. R^2^ values for specific variables arise from analyses with only these regression variables, with subsequent rescaling so that these R^2^ values add to the total regression R^2^. R^2^ values for race/ethnicity pertain to comparisons among the four groups (white, black, Hispanic, other minority).

Suffix C (e.g. BMI-C) = Centered values; for psychosocial & meals at home variables: medium and high are compared to low.

**Table 6:** **Protein, regression of log(biomarker) on log(self-report) and other predictors in NPAAS (n = 450) and R**^**2**^

	**Food frequency questionnaire (FFQ)**			**4 day food record**			**24 Hr diet recall**		
**Variable**	**β**	**SE**	**R**^**2**^	**β**	**SE**	**R**^**2**^	**β**	**SE**	**R**^**2**^
Intercept	4.252*	0.055		4.206*	0.051		4.250*	0.052	
Log(Protein-FFQ-C)	0.132*	0.032	9.295	0.462*	0.049	22.141	0.397*	0.050	16.463
BMI-C	0.009*	0.003	5.481	0.009*	0.003	4.823	0.010*	0.003	5.513
Age-C	-0.012*	0.002	4.03	-0.01*	0.002	2.493	-0.012*	0.002	3.530
Race:			2.65			3.115			3.547
Black	-0.127*	0.043		-0.138*	0.038		-0.152*	0.039	
Hispanic	-0.122*	0.044		-0.10*	0.040		-0.106*	0.041	
Other	-0.029	0.080		0.001	0.073		-0.033	0.075	
Meals at Home:			0.284			0.385			0.337
Med	0.027	0.035		0.046	0.032		0.033	0.033	
High	0.049	0.036		0.049	0.033		0.046	0.034	
Body Image:			0.136			0.254			0.117
Med	0.005	0.042		0.006	0.038		-0.007	0.039	
High	0.040	0.068		0.062	0.063		0.031	0.065	
TFEQ-R18:									
Restraint			0.06			0.077			0.048
Med	-0.016	0.033		-0.020	0.030		-0.011	0.031	
High	0	0.040		0.007	0.037		0.023	0.038	
Unc. Eat.:			0.919			0.608			0.640
Med	-0.065	0.037		-0.059	0.034		-0.050	0.035	
High	-0.068	0.043		-0.048	0.040		-0.055	0.041	
Emo. Eat.:			0.908			1.267			0.789
Med	0.054	0.037		0.062	0.034		0.049	0.035	
High	-0.024	0.050		-0.032	0.046		-0.023	0.047	
Social Desirability:			0.109			0.130			0.112
Med	0.017	0.034		0.007	0.032		0.024	0.032	
High	0.027	0.037		0.029	0.034		0.021	0.035	
Total^1^			23.872			35.291			31.097

BMI, body mass index.

^*^β Coefficient differs from zero at p = 0.05 significance level.

^1^Total – percent of variation explained by all variables. R^2^ values for specific variables arise from analyses with only these regression variables, with subsequent rescaling so that these R^2^ values add to the total regression R^2^. R^2^ values for race/ethnicity pertain to comparisons among the four groups (white, black, Hispanic, other minority).

Suffix C (e.g. BMI-C) = Centered values; for psychosocial & meals at home variables: medium and high are compared to low.

**Table 7:** **Protein density, regression of log(biomarker) on log(self-report) and other predictors in NPAAS (n = 450) and R**^**2**^

	**Food frequency questionnaire**			**Four day food record**			**24 Hr diet recall**		
**Variable**	**β**	**SE**	**R**^**2**^	**β**	**SE**	**R**^**2**^	**β**	**SE**	**R**^**2**^
Intercept	2.602*	0.057		2.629*	0.055		2.659*	0.058	
Log(PD)C	0.325*	0.073	6.254	0.511*	0.071	12.416	0.427*	0.074	8.578
BMI-C	-0.004	0.003	0.66	-0.004	0.003	0.658	-0.004	0.003	0.448
Age-C	-0.003	0.003	0.06	-0.001	0.002	0	-0.004	0.003	0.222
Race:			2.396			2.988			3.323
Black	-0.107*	0.043		-0.129*	0.041		-0.138*	0.042	
Hispanic	-0.101*	0.045		-0.080	0.044		-0.092*	0.045	
Other	-0.042	0.081		-0.005	0.078		-0.060	0.080	
Meals at Home:			0.413			0.189			0.182
Med	0.044	0.036		0.039	0.034		0.031	0.035	
High	0.055	0.037		0.038	0.035		0.039	0.037	
Body Image:			0.669			0.664			0.664
Med	0.043	0.043		0.055	0.042		0.031	0.043	
High	0.109	0.071		0.102	0.068		0.073	0.071	
TFEQ-R18									
Restraint:			0.209			0.234			0.234
Med	-0.030	0.034		-0.030	0.032		-0.026	0.033	
High	-0.023	0.041		-0.019	0.040		0.005	0.041	
Unc. Eat.:			0.319			0.439			0.439
Med	-0.052	0.038		-0.062	0.037		-0.055	0.038	
High	-0.061	0.044		-0.055	0.043		-0.074	0.043	
Emo. Eat.:			0.561			0.605			0.605
Med	0.057	0.038		0.056	0.037		0.057	0.038	
High	0.032	0.052		0.014	0.050		0.020	0.051	
Social Desirability:			0.189			0.524			0.524
Med	-0.012	0.035		-0.029	0.034		-0.016	0.035	
High	0.021	0.038		0.027	0.036		0.032	0.037	
Total^1^			11.731			18.707			14.729

BMI, body mass index.

^*^β Coefficient differs from zero at p = 0.05 significance level.

^1^Total – percent of variation explained by all variables. R^2^ values for specific variables arise from analyses with only these regression variables, with subsequent rescaling so that these R^2^ values add to the total regression R^2^. R^2^ values for race/ethnicity pertain to comparisons among the four groups (white, black, Hispanic, other minority).

Suffix C (e.g. BMI-C) = Centered values; for psychosocial & meals at home variables: medium and high are compared to low.

**Table 8:** **All dietary self-reports: a) energy b) protein c) protein density, regression of log(biomarker) on log(self-report) and other predictors in NPAAS (n = 450) and R**^**2**^

**Variable**	**β**	**SE**	**R**^**2**^	**β**	**SE**	**R**^**2**^	**β**	**SE**	**R**^**2**^
Intercept	7.614*	0.028		4.203*	0.051		2.649*	0.056	
Log(E/P/PD FFQ-C)	0.018	0.019	3.920	0.009	0.032	9.557	0.049	0.084	5.918
Log(E/P/PD 4DFR-C)	0.156*	0.038	5.896	0.353*	0.061	14.545	0.393*	0.090	7.066
Log(E/P/PD 24Hr-C)	-0.006	0.034	0.204	0.189*	0.060	1.551	0.180*	0.090	0.956
BMI-C	0.013*	0.001	26.722	0.008*	0.003	4.112	-0.004	0.003	0.731
Age-C	-0.009*	0.001	8.130	-0.010*	0.002	2.502	-0.002	0.003	0.019
Race:			1.049			2.421			2.891
Black	-0.019	0.021		-0.132*	0.039		-0.131*	0.041	
Hispanic	-0.054*	0.022		-0.085*	0.040		-0.079	0.044	
Other	-0.035	0.039		-0.001	0.072		-0.028	0.079	
Meals at Home:			0.204			0.298			0.122
Med	-0.018	0.017		0.043	0.032		0.034	0.034	
High	-0.003	0.018		0.041	0.033		0.030	0.036	
Body Image:			0.271			0.315			0.467
Med	-0.026	0.021		0.003	0.038		0.047	0.042	
High	-0.039	0.034		0.067	0.062		0.089	0.069	
TFEQ-R18:									
Restraint:			0.257			0.051			0.177
Med	0.021	0.016		-0.014	0.030		-0.029	0.032	
High	0.030	0.019		0.019	0.037		-0.002	0.040	
Unc. Eat:			0.540			0.363			0.365
Med	-0.020	0.018		-0.042	0.034		-0.054	0.037	
High.	-0.023	0.021		-0.037	0.039		-0.056	0.043	
Emo. Eat:			0.532			1.083			0.520
Med	0.014	0.018		0.054	0.033		0.052	0.037	
High	-0.023	0.024		-0.034	0.045		0.015	0.050	
Social Desirability:			0.176			0.125			0.503
Med	0.018	0.017		0.014	0.031		-0.031	0.034	
High	0.007	0.018		0.029	0.034		0.023	0.036	
Total^1^			47.902			36.922			19.733

BMI, body mass index.

^*^β Coefficient differs from zero at p = 0.05 significance level.

^1^Total – percent of variation explained by all variables. R^2^ values for specific variables arise from analyses with only these regression variables, with subsequent rescaling so that these R^2^ values add to the total regression R^2^. R^2^ values for race/ethnicity pertain to comparisons among the four groups (white, black, Hispanic, other minority).

Suffix C (e.g. BMI-C) = Centered values; for psychosocial & meals at home variables: medium and high are compared to low.

Log E/P/PD stands for E = Energy for corresponding a) Energy data; P = Protein for corresponding b) Protein data; PD = Protein Density for corresponding c) Protein Density data.

## Discussion

In this study, we find that the addition of psychosocial variables had a small contribution to improving the recovery of the variation in short-term energy and protein consumption based on biomarkers in a sample of post-menopausal US women. However, as evidenced in the OPEN study, the amount of variation explained by psychosocial predictors of energy underreporting is relatively low [11]. Overall these women score highly on all dimensions of the TFEQ, especially in uncontrolled eating; social desirability scores are also high. Similar women (mean age: 55.6 y+/-12.7 y; n = 919) in the United Kingdom scored lower on the TFEQ scale (13.4+/-3.6 for cognitive restraint; 16.1+/-4.8 for uncontrolled eating; 6.4+/-2.8 for emotional eating) [22]. Whether this finding is a by-product of a society that is more accepting and more likely to acknowledge “uncontrolled eating” in the U.S. as opposed to the United Kingdom or whether other variables are at play needs to be more fully explored. The finding that women eating more meals at home are less likely to under-estimate their FFQ energy is important as that implies the importance of the home context in helping women remember what they ate perhaps because they were involved in preparing the food. The body image discordance variable is similar to that reported for US women aged 40–69 y (n = 223 women; under-reporters: 1.33+/0.10 and accurate reporters: 1.1+/-0.09) [11]

The high mean social desirability score of 21.7+/-5.35 is analogous to that of female adoption applicants (22.6+/-5.6) [24] suggesting that NPAAS women present themselves in a highly socially desirable light. Perhaps this finding is a reflection of the positive attributes of a group of women who volunteered for a study that has high participant burden.

With respect to recovery of biomarker variation all psychosocial factors provide quite modest contribution. Components of the psychosocial variables contributing to recovery of biomarker variation for protein and protein density include emotional eating and body image discordance; for energy intake emotional eating contributes the most. Women with high social desirability or body image discordance or emotional eating also under-report protein foods possibly because they consider it a “low value” food and may associate it with unhealthy foods such as hot dogs and red meat. Additional research on why the reporting of protein and energy are impacted in different ways by psychosocial variables in this sample of post-menopausal women is warranted.

An interesting finding is the differential reporting of protein not only by diet assessment tool, but also by participant characteristics such as ethnicity. For example protein as assessed by FFQ is under-reported which could be related to inadequate listing of protein foods familiar to this sample of diverse post-menopausal women. However, the opposite that is over-estimation of protein, is evidenced when diets of Black participants are assessed via the 24HR or 4DFR. Avenues to improve dietary assessment include encouraging the dietary recall interviewers not to over-probe protein intake. FFQs can also be improved by ensuring that the food list is sufficiently representative of protein foods and foods consumed overall by the sample under study. An additional finding is that protein intake is under-reported to a lesser degree than energy intake, and the contribution to variance in self-report of protein intake is not explained as much by body size or age, as is the case for energy (R^2^ of 26.7% and 8.1% for energy vs. 4.1% and 2.5% respectively for protein, body size and age respectively). Perhaps other factors such as psychosocial and cultural factors play a larger role in the reporting of protein.

Curiously in this study social desirability was predictive of under-reporting in the FFQ, but not 24HR, contrary to results in the OPEN study [11]. It is possible that this finding could relate to improvements in training the 24HR interviewer so that he or she did not motivate participants to respond in a socially desirable manner as intimated by Tooze et al. in the OPEN study [11]. Interestingly the impact of restraint of eating was not significant in helping explain biomarker variance.

In deciding whether to apply psychosocial scales in a measurement error study, it would be helpful to understand the socio-cultural background of the sample under question. For example in samples of new immigrants an acculturation scale may serve as a useful predictor of variation in energy or protein intake as it captures the degree with which the attributes of the mainstream culture that affect intake such as body image ideals are assimilated. Limitations of this study include the less than optimal matching of the time frame covered by self-report measures and the biomarker measurement. For example the 24HR were conducted at monthly intervals after the biomarker measurements; however, the 4DFR collection corresponds to the two week period when the DLW was expended and the WHI-FFQ assessed intake in the three month period prior to visit one. As for protein, only one 24 hour sample was collected before visit two; the closest dietary measure to this time point is the four day food record. The related assumption in comparing self-report instruments is that the day-to-day energy and protein intake of post-menopausal women who are weight stable is not highly variable. Note that for disease association analyses, biomarker-calibrated consumption estimates can be used as quantitative variables, or categorized into consumption quantiles. Other limitations include limited range of variation with respect to social desirability with nominal numbers in the low and majority scoring in the high range which may have contributed to the modest increase in amount of biomarker variance explained by psychosocial variables.

## Conclusions

In summary, the addition of psychosocial and dietary behavior factors to the calibration equations in this sample of post-menopausal women in the US leads to a modest increase in the amount of total biomarker variance explained (R^2^) for energy, protein, and protein density. The contribution of these variables was generally small compared to that of BMI, age and race/ethnicity indicating that calibrated energy and protein consumption estimates based on self-report in conjunction with BMI, age and ethnicity only are likely to be sufficient for most epidemiologic purposes. However for protein consumption estimates psychosocial and diet behavior variables seems to play a larger role. This finding points to the constellation of factors that differentially affect reporting of dietary components. Nevertheless, the study of the psychosocial factors considered here provides valuable additional insight into the dietary reporting practices and provides additional precision in estimates of intake in postmenopausal women in the United States.

## Abbreviations

WHI/NPAAS: Women’s health initiative nutrition & physical activity assessment study; FFQ: Food frequency questionnaire; 24HR: 24 hour dietary recall; 4DFR: Four day food record.

## Competing interests

The authors declare that they do not have competing interests.
